# Successful Treatment of Palmoplantar Psoriasis With Bimekizumab Following Secondary Loss of Response to Brodalumab: A Case Report

**DOI:** 10.7759/cureus.115287

**Published:** 2026-08-27

**Authors:** Priyanka Misra, Nader Ghaderi

**Affiliations:** 1 Dermatology, St. Luke's Hospital, Leeds, GBR

**Keywords:** bimekizumab, biologic therapy, brodalumab, interleukin-17, palmoplantar psoriasis, secondary loss of response to treatment, secondary treatment failure

## Abstract

Palmoplantar psoriasis is a rare variant of psoriasis localised to the palms and soles. The condition accounts for approximately 3%-4% of all psoriasis cases, and disease severity is often underestimated by traditional measures such as the Psoriasis Area and Severity Index (PASI) score. Other assessment tools, including the Palmoplantar Psoriasis Global Assessment (PPPGA) score, are frequently used instead. Topical therapies such as corticosteroids or vitamin D analogues form the basis of initial management. Systemic therapies involving biologic agents are often reserved for more severe cases when previous treatment options have failed.

We report a case of a woman with a 31-year history of palmoplantar psoriasis who had previously trialled topical and systemic therapies with minimal response. After achieving disease control on brodalumab for three years, the patient presented with a flare of palmoplantar psoriasis consistent with treatment failure. By discontinuing brodalumab and initiating bimekizumab, marked improvement in plaque clearance was observed within four months, with no adverse effects.

This case presents secondary loss of efficacy to brodalumab in the treatment of palmoplantar psoriasis, a phenomenon that has not previously been reported. It highlights that even biologics associated with the highest rates of skin clearance are susceptible to loss of efficacy and reinforces the importance of individualising biologic selection to achieve long-term disease control.

## Introduction

Palmoplantar psoriasis is confined to the palms and soles, and although there is no subtype classification, it can present with different morphologic patterns, including scaly, hyperkeratotic plaques as well as pustular lesions [1]. It is well-recognised as being recalcitrant and difficult to treat, given the areas affected tend to be refractory to standard therapies. Difficult-to-treat areas of psoriasis are associated with psychological comorbidities including sleep disturbances and mood disorders. Certain studies also suggest that patients with palmoplantar psoriasis suffer from greater health-related quality-of-life (QOL) impairments than those with moderate to severe plaque psoriasis [2]. Traditionally, a Psoriasis Area and Severity Index (PASI) score is used in patients with psoriasis to assess disease severity at any given time. However, in palmoplantar psoriasis, given the reduced body surface area affected, PASI scores often underestimate disease impact, and hence the Palmoplantar Psoriasis Global Assessment (PPPGA) score is used instead [3]. PPPGA scores rate disease severity on a scale of zero (clear) to four (severe) based on erythema, scaling, pustules, and fissures [4]. We utilized the PPPGA scoring system in this case to allow a standardized assessment of the patient’s disease presentation at baseline and following initiation of treatment.

The management of palmoplantar psoriasis involves a range of therapies, including topical corticosteroids, phototherapy, systemic agents, and biologics. There is currently no clear treatment algorithm for biologic use in palmoplantar psoriasis due to limited evidence [5]. Previous RCTs (GESTURE trial) have suggested secukinumab as having high clearance rates, but further clinical trials are needed to compare the efficacy of different biologic treatment options [6].

Primary and secondary biologic failure can occur in palmoplantar psoriasis, and a range of biologic therapies are used to manage this disease, including ixekizumab, secukinumab, and brodalumab, which all target the IL-23/IL-17 pathway. Brodalumab differs mechanistically from other IL-17 inhibitors by targeting the IL-17 receptor A [7]. It thereby blocks signals from multiple IL-17 family cytokines and is well-documented as being effective in treating recalcitrant psoriasis due to its broad inhibitory profile [8]. In contrast, bimekizumab specifically inhibits both interleukin (IL)-17A and 17-F, providing a potential alternative therapeutic approach for patients who experience loss of efficacy to other IL-17-targeted therapies [5]. We report a rare case of secondary treatment failure to brodalumab in palmoplantar psoriasis, with significant disease clearance following transition to bimekizumab.

## Case presentation

We report the case of an 81-year-old female with a history of chronic palmoplantar psoriasis on a background of rheumatoid arthritis, osteoporosis, osteoarthritis, and a previous transient ischaemic attack. The patient presented to our clinic following a one-month history of active hyperkeratotic plaques with fissuring to both palms and fingertips. The patient’s hands were significantly more affected than her feet, with a noticeable impact on her manual dexterity and inability to manage activities such as holding objects and dressing herself. Although a baseline PPPGA score was not recorded, retrospective analysis of the clinical photographs and documentation would be consistent with a PPPGA score of 4 (severe) at presentation (Figure *1*).

**Figure 1:**
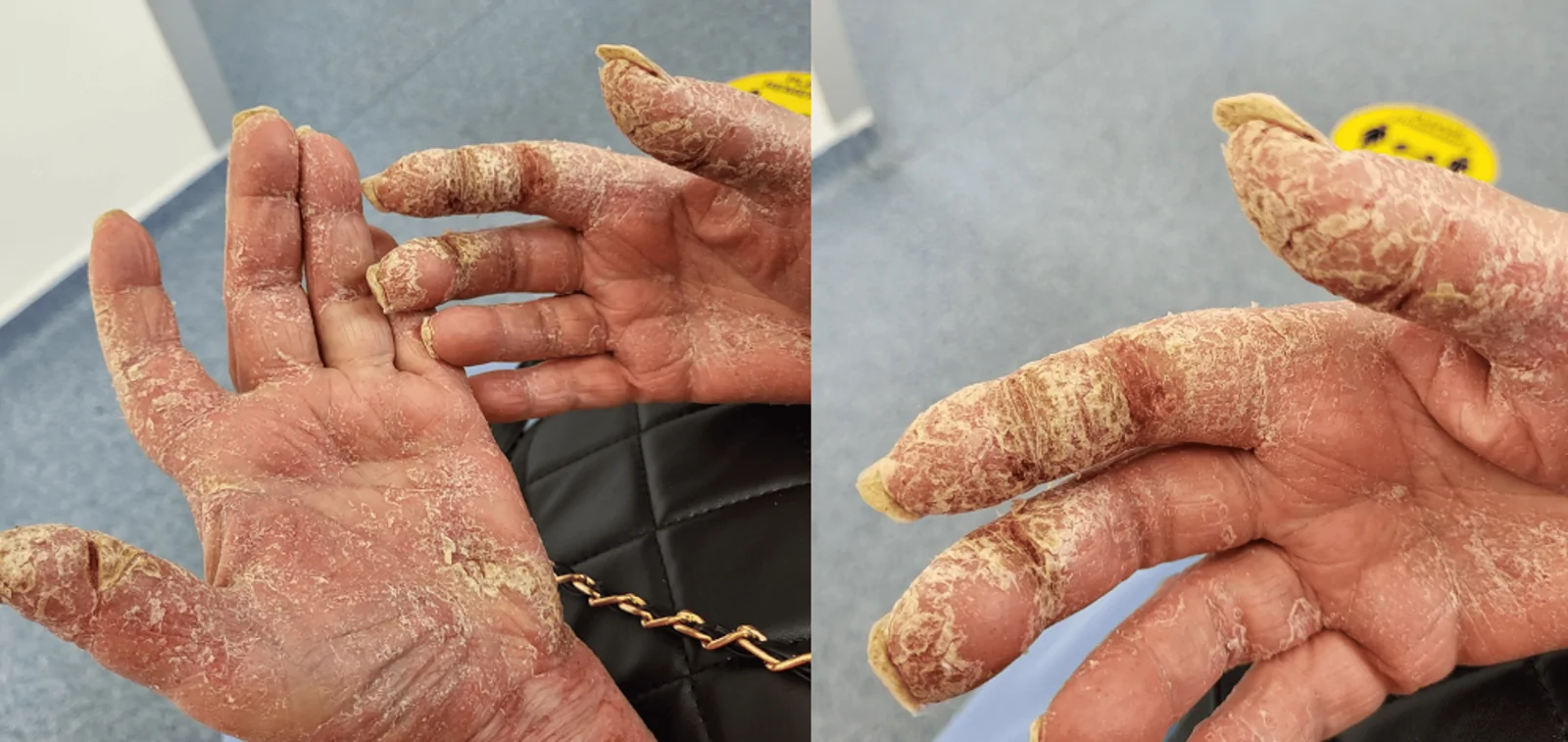
Palmar surfaces prior to treatment with bimekizumab Bilateral erythematous and hyperkeratotic plaques with prominent fissuring are noted to both palms and fingertips alongside subungual hyperkeratosis

Previously trialled treatments for this patient’s palmoplantar psoriasis included methotrexate, ciclosporin, acitretin, adalimumab and ustekinumab. Following non-response to multiple topical and biologic therapies, the patient had been commenced on brodalumab. Up until her presentation to our clinic, the patient achieved disease control for over three years on brodalumab 210mg taken every two weeks alongside emollients, diprosalic ointment and topical corticosteroids, with only one reported flare during this period.

Following her initial review in clinic, the patient was trialled on a two-month course of topical steroids as well as antifungal treatment to cover for a super-added fungal infection. A two-week course of doxycycline was also prescribed with no alteration to her biologic therapy. Five months after her initial clinical review, the patient’s symptoms had shown no improvement with the above interventions. Her palms remained bilaterally erythematous with scaly plaques located across the fingertips alongside worsening subungual hyperkeratosis. Concerns were also raised about the patient’s psychological well-being given her low mood and inability to undergo a knee operation due to poor disease control of her palmoplantar psoriasis. Following this, the decision was then made to discontinue brodalumab and initiate bimekizumab.

Following a four-week washout period, bimekizumab 320 mg every four weeks was commenced after shared decision-making and counselling regarding potential risks and bene­fits. At a follow-up review four months after bimekizumab initiation, the patient demonstrated near-complete resolution of cracking skin to both palms/ fingertips and a significant reduction in the number of plaques. Retrospective review of the follow-up photographs after four months of bimekizumab treatment would be consistent with a PPPGA score of 1 (almost clear) (Figure *2* *and* Table* 1*). The patient was then initiated on the bimekizumab maintenance dose of 320 mg every eight weeks from week 16 onwards. At one-year clinic follow-up, no photographs were taken, but good disease control was reported. The patient had experienced no adverse effects to date and had full restoration of hand function.

**Figure 2:**
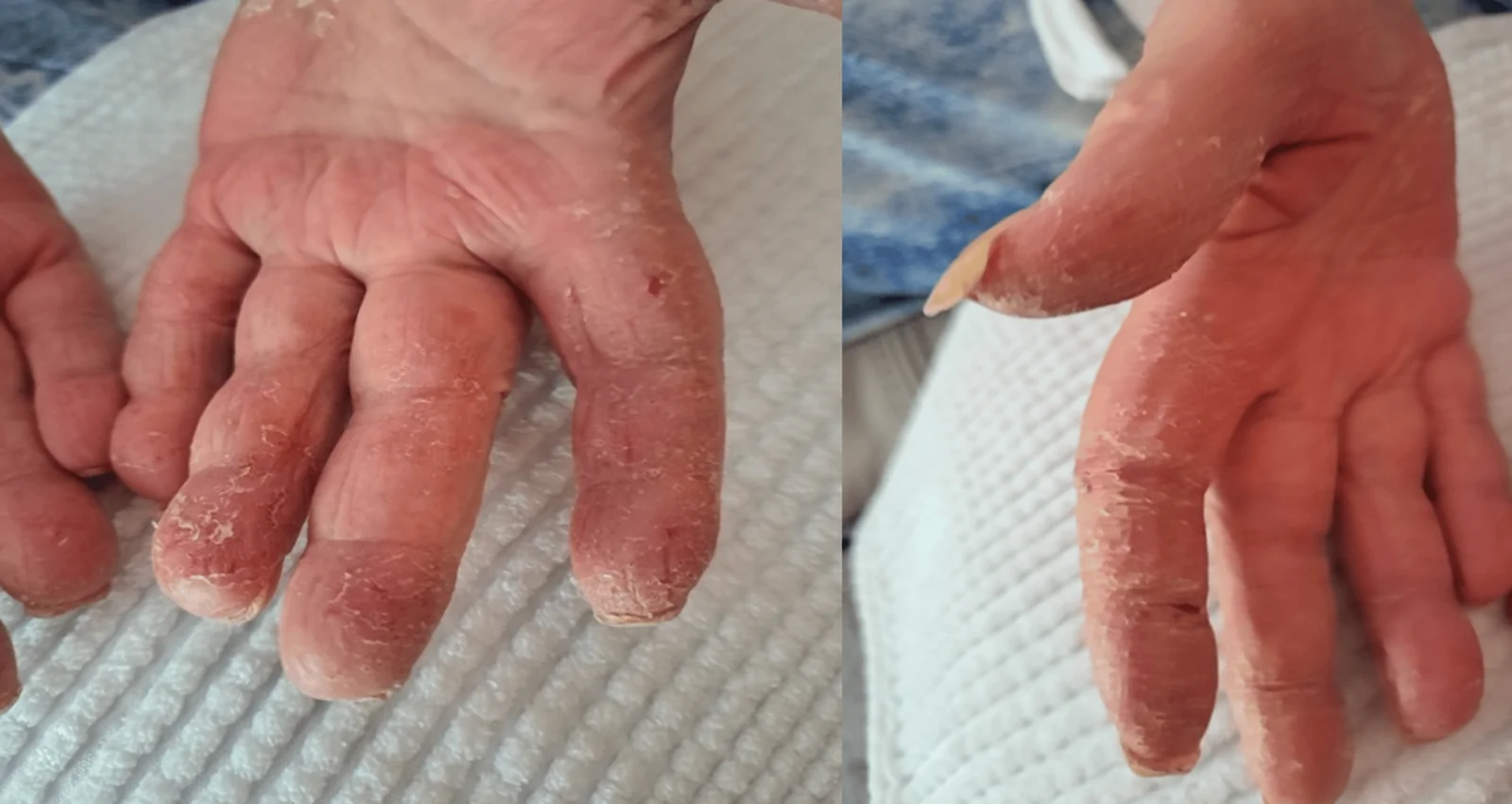
Palmar surfaces following discontinuation of brodalumab and four months of treatment with bimekizumab Marked reduction in the number of hyperkeratotic plaques as well as significant improvement in subungual hyperkeratosis and fissured lesions

**Table 1:** Results table The above table demonstrates the significant improvement in the patient’s Palmoplantar Psoriasis Global Assessment (PPPGA) score after discontinuing brodalumab and completing four months of treatment with bimekizumab

	Palmoplantar Psoriasis Global Assessment (PPPGA) score
Baseline	4 (Severe)
Four months of treatment with bimekizumab	1 (Almost clear)

## Discussion

This case highlights the clinical challenge posed by secondary biologic failure when treating palmoplantar psoriasis. Secondary loss of biologic response has been previously attributed to factors such as metabolic comorbidities, immunogenicity, and a history of biologic use. A shift in disease pathophysiology where alternative cytokine pathways become involved has also been linked to secondary biologic failure [9]. The efficacy of brodalumab in maintaining long-term disease control is also challenged by this case and reinforces a need for further evidence regarding switching between IL-17 pathway inhibitors. To our knowledge, this is the first reported case of successful treatment with bimekizumab following secondary loss of efficacy to brodalumab in palmoplantar psoriasis.

Brodalumab is a human IL-17 receptor A antagonist, thereby inhibiting multiple signals of the IL-17 family cytokines [7]. Although no dedicated clinical trials have evaluated brodalumab in palmoplantar psoriasis, efficacy in treating plaque psoriasis with previous biologic failure has been noted. In a subgroup analysis of the AMAGINE-2/3 trial, patients in the ustekinumab group with inadequate response who then switched to broadlumab showed an increase in PASI 75% improvement from baseline from week 12 to week 52 [10]. Additionally, a case series by Politou et al. assessed treatment initiation with brodalumab in patients who failed prior treatment with secukinumab and revealed that 100% of patients with palmoplantar pustulosis achieved complete skin clearance by the 16th week [11].

Although our patient maintained disease control with brodalumab for three years, this case suggests that secondary loss of efficacy may occur despite an initially sustained response. This highlights that even biologics associated with a broad inhibitory profile and high rates of skin clearance are susceptible to treatment failure. Given the limited evidence available for palmoplantar psoriasis, further reports of secondary biologic failure as well as prospective studies evaluating switching strategies are needed to inform long-term management.

Evidence assessing the efficacy of bimekizumab in palmoplantar psoriasis remains limited. Bimekizumab is a biologic therapy that specifically inhibits both IL-17A and IL-17F. A recent meta-analysis comparing the efficacy of eight biologic therapies in achieving disease clearance in palmoplantar psoriasis revealed that bimekizumab was the second most effective [12]. However, evidence regarding the efficacy of bimekizumab following previous biologic failure in palmoplantar psoriasis remains scarce. Our findings from this case are further supported by a recent case report in 2025 documenting the near-complete clearance of symptoms at one-month follow-up in a patient with palmoplantar psoriasis who had previous biologic non-response to secukinumab and adalimumab [13]. Together, these reports may reflect differences in the mechanisms of IL-17 inhibition and suggest that failure of one IL-17-targeted biologic should not necessarily preclude response to another agent acting within the same inflammatory pathway. While brodalumab blocks IL-17 receptor A and therefore inhibits signalling from multiple IL-17 cytokines, bimekizumab directly neutralises both IL-17A and IL-17F. Dual IL-17A/F inhibition has been shown to result in profound suppression of psoriatic inflammatory and keratinocyte responses [14]. Although both agents ultimately inhibit IL-17A/IL-17F signalling, their distinct mechanisms of action may result in differences in the pattern or extent of pathway suppression, potentially contributing to the response to bimekizumab observed in our patient.

Nevertheless, our case was limited by retrospective PPPGA scoring. By not having prospective measurements, this introduced the potential for observer bias and potential discrepancies in PPPGA scores at baseline and four-month follow-up. Additionally, the follow-up photographs were taken only four months after bimekizumab initiation. Although good disease control was documented at one-year clinic review, the absence of photographic data limits the reliability with which we can assess the true long-term benefits of bimekizumab in recalcitrant palmoplantar psoriasis. Larger prospective studies are required to determine the optimal sequencing of biologic therapies in this disease subset and truly assess whether bimekizumab represents a valuable treatment option for patients with recalcitrant palmoplantar psoriasis who develop secondary loss of efficacy to other IL-17 inhibitors.

## Conclusions

Palmoplantar psoriasis can cause substantial functional and psychological morbidity despite affecting a relatively small body surface area. This case describes secondary loss of response following approximately three years of successful brodalumab therapy, followed by a near-complete disease clearance and restoration within four months of initiating bimekizumab. 

The observed response suggests that failure of one IL-17 pathway inhibitor does not necessarily preclude benefit from another agent with a different mechanism of action within the same inflammatory pathway. Bimekizumab may represent a valuable therapeutic option for patients with recalcitrant palmoplantar psoriasis who experience secondary loss of response to brodalumab. Given that this is a single case report, the findings cannot be directly applied to clinical practice, and prospective comparative studies with longer-term follow-up are required to clarify the optimal sequencing of biologic therapies in this difficult-to-treat patient population. 
